# CELLULAR IMMUNE AGING AND PHYSICAL DISABILITY: RESULTS FROM THE HEALTH AND RETIREMENT STUDY

**DOI:** 10.1093/geroni/igad104.1007

**Published:** 2023-12-21

**Authors:** Grace Noppert, Kate Duchowny

**Affiliations:** University of Michigan, Ann Arbor, Michigan, United States; University of Michigan, Ann Arbor, Michigan, United States

## Abstract

Given long-standing inequities in physical disability, we leveraged data from the nationally-representative Health and Retirement Study to investigate gender differences in the association between advanced immune aging and physical disability. Among women, we found a one standard deviation increase in the CD8+:CD4+ ratio, a marker of advanced immune aging, was associated with a 24% greater prevalence of ADL disability (PR= 1.24, 95% CI: 1.23, 1.25). No association was observed among men. Results suggest that among women, advanced immune aging may serve as a salient risk factor in shaping physical disability.

